# The relationship between educational level and bone mineral density in postmenopausal women

**DOI:** 10.1186/1471-2296-5-18

**Published:** 2004-09-06

**Authors:** Ali Gur, Ayşegül Jale Sarac, Kemal Nas, Remzi Cevik

**Affiliations:** 1 Physical Medicine and Rehabilitation, Medical Faculty, Dicle University, Diyarbakir, Turkey; 2 Physical Medicine and Rehabilitation, Medical Faculty, Dicle University, Diyarbakir, Turkey; 3 Physical Medicine and Rehabilitation, Medical Faculty, Dicle University, Diyarbakir, Turkey

## Abstract

**Background:**

This study describes the influence of educational level on bone mineral density (BMD) and investigating the relationship between educational level and bone mineral density in postmenopausal women.

**Methods:**

A total of 569 postmenopausal women, from 45 to 86 years of age (mean age of 60.43 ± 7.19 years) were included in this study. A standardized interview was used at the follow-up visit to obtain information on demographic, life-style, reproductive and menstrual histories such as age at menarche, age at menopause, number of pregnancies, number of abortions, duration of menopause, duration of fertility, and duration of lactation. Patients were separated into four groups according to the level of education, namely no education (Group 1 with 209 patients), elementary (Group 2 with 222 patients), high school (Group 3 with 79 patients), and university (Group 4 with 59 patients).

**Results:**

The mean ages of groups were 59.75 ± 7.29, 61.42 ± 7.50, 60.23 ± 7.49, and 58.72 ± 7.46, respectively. Spine BMD was significant lower in Group 1 than that of other groups (p < 0.05). Trochanter and ward's triangle BMD were the highest in Group 4 and there was a significant difference between Group 1 and 4 (p < 0.05). The prevalence of osteoporosis showed an inverse relationship with level of education, ranging from 18.6% for the most educated to 34.4% for the no educated women (p < 0.05). Additionally, there was a significant correlation between educational level and spine BMD (r = 0.20, p < 0.01), trochanter BMD (r = 0.13, p < 0.01), and ward's BMD (r = 0.14, p < 0.01).

**Conclusions:**

The results of the study suggest that there is a significant correlation between educational level and BMD. Losses in BMD for women of lower educational level tend to be relatively high, and losses in spine and femur BMD showed a decrease with increasing educational level.

## Background

Osteoporosis has recently been recognized as a major public health problem by some governments and health care providers. In the European community, the number of men and women aged 65 years of older will increase steadily and the most dramatic changes will occur in the very elderly, in whom the incidence of osteoporotic fracture is greatest [1]. As the populations gets older, morbidity, mortality and financial costs attributed to osteoporosis are expected to rise. The economic costs related to osteoporotic fractures are substantial and will almost certainly increase further unless effective preventive interventions are widely implemented [2].

Peak bone mass is achieved soon after puberty, and bone is lost with various "insults", including ageing and postmenopausal changes. Factors influencing peak bone mass and loss range from nutrition, to lifestyle, to certain medical disorders. Educational level may also have an effect on bone mineral density since there is relationship between educational level and reproductive factors such as pregnancy and lactation and other lifestyle factors [3-7].

In developed countries a higher prevalence of most chronic diseases has been recognized among lower socio economic levels and in less educated subjects [8-14]; however, only a few and conflicting data are available for osteoporosis [15-18].

Since many risk factors for osteoporosis, such as diet, deficiency of trace minerals, reproductive factors, inactivity and tobacco use, are lifestyle variables related to social and cultural background [18-25], the influence of formal educational level on bone mineral density [BMD], together with establishment of a relationship between formal educational level and bone mineral density in postmenopausal women are the main concern of this study.

Patients were compared according to years of formal education. We used formal education because it may be regarded as a composite or surrogate variable for overall socioeconomic status [10], and level of education [years of completed education) allows comparison between countries more readily than other socioeconomic indicators [13].

Purpose of this study was to evaluate the influence of formal education on BMD and investigating the relationship between educational level and bone mineral density in the postmenopausal women.

## Methods

In Department of Physical Medicine and Rehabilitation, of the total 701 consecutive women screened, 132 were excluded. This study was undertaken in Dicle University, Diyarbakir, Turkey. The study protocol was reviewed and approved by the Dicle University Ethics Committee, and informed consent was obtained from all participants. A detailed history was taken from each woman including relevant life-style parameters and risk factors, and their weight and height measurements were recorded. The following exclusion criteria were applied for further analyses: (1) fractures after the age of 25; (2) menopause before the age of 40; (3) amenorrhoea greater than 6 months; (4) chronic conditions affecting bone density; (5) any use of corticosteroids.

A total of 569 postmenopausal women, at 45 to 86 years of age (mean age of 60.43 ± 7.19 years) were considered. BMD of the spine and hip (neck, trochanter, and ward's triangle) were measured by dual-energy x-ray absorptiometry (NORLAND, 6938CE, New York, USA). According to the WHO [26] osteoporosis was defined as a lumbar BMD value more than 2.5 SD below the T-score, corresponding to 0.759 g/cm^2 ^[27]. The variation coefficient for consecutive determinations on spine and femur images in our laboratory was 1.9% at the lumbar spine and 1.6 % at the femur region. All spinal scans were reviewed for evidence of vertebrae with collapse or focal sclerosis by an experienced radiologist.

In order to standardize the procedure, the patients all answered the same specially developed questionnaire supervised by the doctor (revised from the MEDOS Form) [28]. A standardized interview was used at the follow-up visit to obtain information on demographic, life-style, reproductive and menstrual histories such as age at menarche, age at menopause, number of pregnancies, number of abortions, duration of menopause, duration of fertility, and duration of lactation.

The level of education is categorized in four groups according to the number of school years and the highest qualification received; no education (Group 1 = 209 patients), elementary (8 years or less, Group 2 = 222 patients), high school (9–11 years, Group 3 = 79 patients), and university (12 years or more, Group 4 = 59 patients). Body mass index (BMI; weight / height^2^) was obtained through height and weight measurements by using a wall-mounted ruler and a digital scale.

Recent dietary calcium intake (past 12 months) was assessed using standardized food models to estimate portion sizes [24]. Dietary calcium intakes were analyzed in two groups as inadequate (<500 mg/day) and adequate (500–1000 mg/day) [25]. The number of drinks consumed per week in the past 30 days, was used as the measure of current alcohol consumption (never use, very rare, frequently). Women who had smoked at least ten cigarettes per day during the five postmenopausal years were classified as smokers [25]. All patients classified, in terms of their reported current and life long smoking, into such group: 1) never use, 2) less than 1 packet, 3) 1–2 packet, and 3) more than 2 packets per day. They were also classified, in terms of their reported current and life-long caffeine use, into such groups: 1) never use, 2) 2 or below cup caffeinated coffee per day, 3) 3 or above cups caffeinated coffee per day. Physical activity is assessed by inquiring about the reported number of 20-min sessions of leisure-time physical activity per week and physically active behavior is defined as participation in more than two sessions per week; job-related physical exercise is not taken into account.

### Statistical analyses

The statistical analyses were carried out with the SPSS/ PC-program. Differences in proportions for categorical variables were tested by chi-square test. The data are expressed as means ± SD. Statistical significance was tested using one way-ANOVA test and post-hok Bonferroni test for comparison of different groups. Pearson correlation test were computed to measure the association between the variables studied. The statistical significant set if the p-value was less than 0.05.

## Results

Their reproductive and demographic characteristics are shown in Table 1. When comparing the adequate calcium intake, the most educated women showed a statistically significant higher percentage than that of the other groups (p < 0.05). The mean ages of groups were 59.75 ± 7.29, 61.42 ± 7.50, 60.23 ± 749, and 58.72 ± 7.46, respectively. There was no significant difference among all groups with respect to age, BMI, age at menarche, age at menopause, and duration of menopause (p > 0.05) (Table 2).

**Table 1:** Values for various reproductive and personal characteristics among 569 women screened for osteoporosis according to education level

**Variable**	**No Education (Group 1 = 209)**	**Elementary (Group 2 = 222)**	**High school (Group 3 = 79)**	**University (Group 4 = 59)**
**Menstrual cycle pattern, (%)**				
Regular cycles	159 (76)	151 (68)	66 (84)	43 (73)
Irregular cycles	50 (24)	71 (22)	13 (16)	16 (27)
**Menopausal status, (%)**				
Natural	171 (82)	189 (85)	72 (91)	40 (69)
Iatrogenic	38 (18)	33 (15)	7 (9)	19 (31)
**Premenopausal HRT, (%)**				
Never use	195 (93)	195 (88)	59 (75)	51 (86)
Ever use	14 (7)	27 (12)	20 (25)	8 (14)
**Postmenopausal HRT, (%)**				
Never use	201 (96)	203 (91)	63 (79)	52 (88)
Ever use	8 (4)	19 (9)	16 (21)	7 (12)
**Physical daily activity, (%) Childhood**				
Inactivity	6 (3)	33 (15)	14 (18)	8 (14)
Mild activity	100 (48)	118 (53)	59 (75)	32 (54)
Serious activity	103 (49)	71 (32)	6 (7)	19 (32)
**Adolescent**				
Inactivity	8 (4)	35 (16)	15 (19)	9 (15)
Mild activity	60 (29)	105 (47)	54 (68)	19 (32)
Serious activity	141 (67)	82 (37)	10 (13)	31 (53)
**Adult**				
Inactivity	56 (27)	83 (37)	38 (48)	22 (37)
Mild activity	94 (45)	97 (44)	36 (46)	21 (36)
Serious activity	59 (28)	42 (19)	5 (6)	16 (26)
**Calcium intake, (%)**				
Adequate	102 (49)	107 (48)	36 (46)	45 (76)*
Inadequate	107 (51)	115 (52)	43 (54)	14 (24)
**Smoking, (%)**				
Never use	203 (97)	185 (83)	59 (75)	40 (69)
< 1 packet	4 (2)	18 (8)	6 (7)	9 (15)
1–2 packet	2 (1)	9 (4)	10 (13)	1 (2)
> 2 packet	-	10 (5)	4 (5)	8 (14)
**Coffee, (%)**				
Never use	117 (56)	102 (46)	43 (54)	20 (34)
2 or below cup	88 (42)	85 (38)	20 (25)	23 (39)
3 or above cup	4 (2)	35 (16)	16 (21)	16 (26)
**Alcohol, (%)**				
Never use	203 (97)	204 (92)	64 (81)	40 (68)
Very rare	2 (1)	12 (5)	10 (13)	14 (24)
Frequently	4 (2)	6 (3)	5 (6)	5 (8)

*Significant different from other groups (p < 0.05).

**Table 2:** Comparison of reproductive characteristics of 569 women according to education level

	**No Education**	**Elementary**	**High school**	**University**
Age (years)	59.75 ± 7.29	61.42 ± 7.50	60.23 ± 7.49	58.72 ± 7.46
Body Mass Index	27.18 ± 5.14	28.23 ± 5.63	27.72 ± 6.14	28.45 ± 5.56
**Age at menarche (years)**	13.77 ± 1.36	13.75 ± 1.28	14.09 ± 0.83	13.22 ± 1.38
**Age at menopause (years)**	45.26 ± 5.57	45.22 ± 6.72	47.25 ± 4.69	45.38 ± 5.25
**Duration of fertility (years)**	31.48 ± 5.73	31.47 ± 6.79	33.25 ± 4.81	32.16 ± 5.61
**Duration of menopause (years)**	14.49 ± 7.8	13.20 ± 8.17	12.02 ± 8.02	12.41 ± 8.73
**Number of abortions**	1.31 ± 1.51^b, c^	0.94 ± 1.64	0.51 ± 1.17	0.19 ± 0.54
Number of pregnancies	7.11 ± 3.38^a, b, c^	4.93 ± 3.61^d, e^	2.27 ± 1.91	2.29 ± 1.39
**Duration of lactation (months)**	133.2 ± 54.3^a, b, c^	93.62 ± 50.66^d, e^	45.76 ± 38.83	57.77 ± 35.36

Values are shown as mean ± standard deviation (SD) for all variables.

^a^Significant different from elementary group, ^b ^Significant different from high school group, ^c^Significant different from university group, ^d ^Significant different from high school group, and ^e ^Significant different from university group (p < 0.05).

Number of abortions was higher in group 1 and 2 than those of group 3 and 4 (p < 0.05). There was no significant difference with respect to number of pregnancies and duration of lactation between group 3 and 4 while there was a significant difference among other groups (p < 0.05), and number of pregnancies and duration of lactation were found to be the highest in Group 1 and 2. Number of pregnancies and duration of lactation in Group 1 were 7.11 ± 3.38 and 133.23 ± 54.34 months and in Group 2 were 4.93 ± 3.61 and 93.62 ± 50.66 months (Table 2).

Spine BMD was significant lower in Group 1 than that of other groups (p < 0.05). Trochanter and ward's BMD were the highest in Group 4 and there was a significant difference between Group 1 and 4 (p < 0.05). The prevalence of osteoporosis showed an inverse relationship with level of education, ranging from 18.6% for the most educated to 34.4% for the no educated women (p < 0.05) (Table 3).

**Table 3:** Comparisons of bone mineral density of 569 women according to education level

	**No Education**	**Elementary**	**High school**	**University**
**L2-4 BMD**	0.62 ± 0.29^a, b, c^	0.74 ± 0.19	0.77 ± 0.17	0.76 ± 0.33
**Femoral Neck BMD**	0.64 ± 0.19	0.61 ± 0.24	0.62 ± 0.24	0.64 ± 0.21
**Trochanter BMD**	0.49 ± 0.18^c^	0.53 ± 0.19	0.56 ± 0.16	0.61 ± 0.12
**Ward's triangle BMD**	0.45 ± 0.17^c^	0.49 ± 0.18	0.49 ± 0.20	0.58 ± 0.10
**Osteoporosis (%)**	72 (34.4)^a, b, c^	62 (27.9)^d, e^	17 (21.5)	11(18.6)

Values are shown as mean ± standard deviation (SD) for all variables except percentage of osteoporosis.

^a^Significant different from elementary group, ^b ^Significant different from high school group, ^c^Significant different from university group (p < 0.05).

Additionally, there was a significant correlation between educational level and spine BMD(r = 0.20, p < 0.01), trochanter BMD (r = 0.13, p < 0.01), and ward's BMD (r = 0.14, p < 0.01) but wasn't neck BMD (r = -0.02, p > 0.05).

## Discussion

The health care costs, morbidity and mortality excess related to osteoporotic fractures are a major health problem in western countries [29,30]. In order to reduce these medical, social and economic burdens, which are expected to rise in forthcoming years, there is a need for preventive strategies based on health promotion campaigns [31]. To change health behavior related to modifiable risk factors for osteoporosis and to design targeted and more effective health messages [32], the programs have to take into account the socioeconomic and cultural background of the population strata in which the risk for osteoporosis is particularly prominent [15].

Although mechanisms of association between education and osteoporosis remain partly unexplained, most of the risk factors examined have shown distinct trends according to educational level. Although educational level may be an imperfect measure for socio economic status, many studies have clearly established that this marker acts as a good predictor not only for most chronic diseases [10,11,14] but also for many related risk factors [22,23].

In a study by La Vecchia et al., they found that education is a strong determinant of several chronic conditions, and the pattern of health care utilization also varied extensively according to education [11].

Varenna et al. evaluated 6160 postmenopausal women referred for their first densitometric evaluation and they found age at menarche, past exposure to oral contraceptives, prevalence of chronic diseases, physical activity, overweight and smoking showed significant trends according to years of education [15]. Also, as they had a cohort of postmenopausal women as the study group, they could show differences in the prevalence of osteoporosis among educational classes and the protective role played by increases in formal education.

The present study showed that there was no significant difference among all groups with respect to age, BMI, age at menarche, age at menopause, and years since menopause. But there were statistically significant differences among groups in respect to number of pregnancies, duration of lactation, bone mineral density and percentage of osteoporosis.

The comparison with studies performed in other countries can be misleading since eating habits are strongly influenced by ethnic and geographical backgrounds [7]. The meaning of the lower calcium intake observed in the least educated women could be referred to a real difference, taking into account the low sensitivity of the questionnaire used to assess calcium intake. During pregnancy and lactation the growing fetal and neonatal skeletons make major demands for calcium, respectively. There is good evidence now that during lactation a substantial part of this calcium demand is mobilized from the maternal skeleton even despite high dietary calcium. This effect could be especially important with multiple pregnancies and extended lactation.

Magnus et al. undertook a random sample of 1514 Norwegian women and men to investigate knowledge of osteoporosis and attitudes towards methods for preventing this disease, and they concluded that in both men and women, increased knowledge of osteoporosis was correlated to a high level of education [33].

In several studies, authors have found that reproductive history has an inverse relation to bone density [3-6,34-41]. The bone density is adversely affected by both high rate of live birth and long period of breast feeding, common in the region where this study was carried out. The lower birth rate and short period of breast feeding found with the group having university or high school degree, may suggest that both birth rate and the breast feeding period may be associated with educational level. Furthermore, the calcium intake in the group with highest educational level was also found to be considerably higher than that of the other groups. The higher BMD values found with the group of highest educational level, may be attributed to the sufficient amount of calcium intake as was the case with this group

Though the effect of formal educational level on bone mineral density has not yet been well established, the above findings may suggest some hypothetical comments. The findings of this study imply that osteoporosis which is related to bone mineral density, may be related to the educational level and the risks due to higher birth rate, excessive breast-feeding and insufficient calcium intake, and may be controlled through an improvement in educational level.

Because of several limitations, caution must be exercised in interpreting the results of our study. Except for densitometric assessment, the results depend on self-reports. Even though self-report diagnoses have been shown to be valid [6], the level of formal education could bias the report about habits and health practices. Moreover, the sample was not randomly selected and it cannot be considered representative of postmenopausal women in Turkey.

Similar studies are recommended to be carried out in different communities in an effort to confirm whether these findings can be generalized or yield a more complete insight into pathogenetic mechanisms. The knowledge of which population strata may be at greater risk of osteoporosis should be considered carefully for the purpose of health care planning and preventive strategies, making it possible to design tailored and culturally appropriate public health intervention programs.

The protective role played by educational level, which increases with the years of formal education, could be due to other overall determinants can be indirectly inferred from our data, such as a better health status, a more positive attitude to taking drugs and a more efficient use of health care resources. All these determinants can be considered in the light of a greater concern by the women about their own health status, probably related to a different impact of health promotion messages.

## Conclusions

In conclusion, the results of the study suggest that there is a significant correlation between educational level and BMD, and shows differences in the prevalence of osteoporosis among educational classes and the protective role played by increases in formal education. Losses in BMD for women of lower educational level tend to be relatively high, and losses in spine and femur BMD showed a decrease with increasing educational level. Although mechanisms of association between education and low bone mineral density remain partly unexplained, most of the risk factors examined have shown distinct trends according to educational level.

## Competing interests

None declared.

## Financial competing interests

None declared.

## Authors' contributions

AG participated in the design of the study and performed the statistical analyses.

AJS conceived of the study, and participated in its design and coordination.

KN and RC participated in the sequence alignment and screened of subjects.

All authors read and approved the final manuscript.

## Pre-publication history

The pre-publication history for this paper can be accessed here:


